# Monitoring respiratory function with telemedicine devices in asthmatic children

**DOI:** 10.3389/fmed.2025.1604909

**Published:** 2025-06-17

**Authors:** Katalin Kapus, Ferenc Rárosi, Zoltán Novák, Ferenc Peták, József Tolnai

**Affiliations:** ^1^Department of Medical Physics and Informatics, University of Szeged, Szeged, Hungary; ^2^Department of Pediatrics and Pediatric Health Center, University of Szeged, Szeged, Hungary

**Keywords:** pediatric asthma, telespirometry, home spirometry, lung function monitoring, telemedicine

## Abstract

**Introduction:**

Pediatric asthma requires continuous monitoring, traditionally reliant on in-person assessments. Home-based telespirometry offers a promising approach, enabling regular lung function testing, early exacerbation detection, and improved disease management while reducing the burden of in-person visits. However, its effectiveness and accuracy compared to clinical measurements need further evaluation. This study aimed to assess the feasibility and reliability of home spirometry in children with moderate asthma and to compare home-based lung function measurements with those obtained under clinical supervision.

**Methods:**

Eleven children (aged 8–17 years) with moderate asthma were trained to use a handheld spirometer and an associated mobile app. Participants performed home spirometry at least four times per week over a 12-month period, following ERS/ATS standards. Key respiratory parameters, including forced vital capacity (FVC), forced expiratory volume in one second (FEV_1_), FEV_1_/FVC ratio (Tiff), peak expiratory flow (PEF), and forced mid-expiratory flow (FEF_25–75_), were recorded. Data was transmitted to a clinical cloud system for real-time monitoring. Measurement reliability was assessed based on ERS/ATS acceptability criteria, and statistical analyses included mixed ANOVA model, and Bland–Altman analysis with confidence intervals to compare home and clinical measurements.

**Results:**

Home spirometry demonstrated a high rate of reliable measurements, with no significant decline in reliability over time. A positive correlation was observed between the number of home spirometry recordings and the reliability of FEV_1_ and FVC measurements. Comparisons between clinical and first home spirometry measurements showed strong correlations, particularly for FVC. Bland–Altman analyses confirmed good agreement between home and clinical assessments, with narrow limits of agreement for FVC, FEV_1_, and Tiff, whereas PEF and FEF_25–75_ showed greater variability. When expressed as percentage predicted values, similar trends were observed, with FVC% showing the strongest correlation.

**Conclusion:**

The difference in peak flow indices measured at home and lung function labs in asthmatic children highlights the importance of patient education, and the reliabilities indicate the need for frequent assessments. The strong agreement with clinical measurements supports its potential integration into routine asthma care, enabling more accessible and continuous disease management.

## Introduction

Pediatric asthma poses an increasing challenge for global healthcare systems, necessitating innovative approaches to management and monitoring. Conventional care models, primarily reliant on face-to-face consultations, often fall short in effectively addressing the complexities of asthma management in children (1). This inadequacy is underscored by the rising prevalence of asthma exacerbations, which can lead to significant morbidity and increased healthcare utilization (2–4). The Global Initiative for Asthma (GINA) emphasizes that assessing symptom control alone is insufficient, clinicians must also evaluate patients’ risk factors for exacerbations and accelerated lung function decline (5). Consequently, there is a clear need for more effective monitoring strategies, as timely detection of exacerbations is critical for preventing severe outcomes and optimizing treatment (6–8).

Recent advancements in telemedicine have opened a new era for asthma control, particularly through the implementation of telespirometry. This technology allows for regular home lung function testing, enabling patients to monitor their respiratory status without the need for frequent clinic visits (9–11). Studies have shown that home spirometry can enhance patient engagement and provide valuable data for clinicians, facilitating timely interventions in exacerbation scenarios (12–15). The feasibility of telespirometry has been demonstrated in various contexts, including chronic respiratory diseases, where it has proven effective in monitoring lung function and improving patient outcomes (8, 12, 13, 16–18). Furthermore, the integration of digital health interventions has the potential to transform asthma care by providing real-time feedback and personalized management strategies (6, 7, 15).

Recent studies have demonstrated the potential of home spirometry and telemonitoring tools to improve asthma management by enabling regular lung function assessment outside clinical settings. However, most of these earlier studies focused on adult populations (9, 19, 20) or short-term monitoring (21–23), and there remains limited data on long-term feasibility and technical reliability in children. Moreover, variability in study design, measurement standards, and patient selection complicates direct comparisons (10, 14, 18, 23, 24). This underscores the need for further pediatric-focused research that addresses these gaps under real-world conditions.

Our study aims to assess the feasibility of telespirometry specifically in the pediatric population, focusing on the reliability of home measurements. By evaluating the accuracy and consistency of these home-based assessments, we aimed at contributing to the establishment of a framework for integrating telespirometry into routine asthma management (25). This approach not only aligns with current trends in digital health but also addresses the pressing need for more accessible and efficient monitoring solutions for children with asthma (26). As healthcare systems continue to evolve, the incorporation of telemedicine into asthma care could significantly enhance the quality of life for pediatric patients while alleviating some of the burdens on healthcare providers (6, 7, 24, 27).

## Materials and methods

### Participants

The study was approved by the National Institute of Pharmacy and Nutrition, Hungary (No. OGYÉI/8725/2020; address: Hungary, 1051 Budapest, Zrínyi u. 3., dated 16 March 2020) and conducted in accordance with the 1964 Declaration of Helsinki and its amendments. Written informed consent was obtained from all participants, and the trial was registered in the European Union Drug Regulating Authorities Clinical Trials Database under the name *Telemonitoring of Lung Function by Spirometry* (NCT04447664).

**FIGURE 1:**
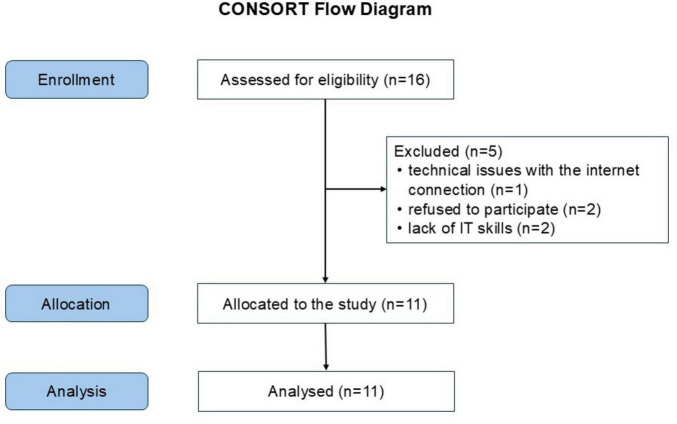
Patient flow chart. A total of 16 participants were assessed for eligibility, meeting criteria including informed consent, being under 18 years old, and the ability to cooperate. Five participants were excluded: one due to internet access issues, two declined participation for personal reasons, and two were ineligible due to insufficient IT skills. The remaining 11 children were enrolled in the study, completed the allocated intervention, and were included in the final analysis.

Participants were required to be under 18 years old with moderate asthma (GINA stages 2–3) (5, 28, 29) and willing to perform home telespirometry as instructed. Exclusion criteria included poor overall health, recent asthma exacerbation requiring clinical care, or any condition deemed unsuitable by the investigator. Participants could withdraw at any time. The study adhered to CONSORT guidelines, with the patient flow chart presented in Figure 1. Of 16 eligible participants, five were excluded: one due to internet access issues, two declined participation for personal reasons, and two were ineligible for using IT tools.

### Spirometry

#### Measurements

Children with moderate asthma (*n* = 11, aged 8–17 years) were trained to use a handheld spirometer (Uscom SpiroSonic Ultrasonic Spirometer, Uscom Europe, Budapest, Hungary) at the Department of Pediatrics and Pediatric Healthcare Center, University of Szeged. The spirometer is a factory-calibrated device that requires no manual recalibration and is clinically approved and certified by international regulatory bodies, including the FDA and CE.

After training, respiratory function parameters were measured in the clinic under specialist supervision. Participants then used the spirometer at home for one year, performing measurements at least four times per week per ERS/ATS standards (30). More frequent measurements were encouraged during periods of asthmatic symptoms. Additionally, an asthma control test, integrated into the app, was completed at least weekly, preferably after each spirometry session.

Before each measurement session, the app automatically performed a zero-flow calibration. The child was then prompted to carry out the prescribed spirometry maneuvers. After each attempt, the app validated the technical quality of the effort and displayed the resulting spirometry curves and numerical parameters. Once three acceptable and reproducible measurements were obtained, the full dataset, including all calculated values and flow-volume loops, was automatically uploaded to a secure clinical database. In cases where participants did not complete the recommended number of weekly measurements, the assistance team contacted the parents to inquire about the cause. This follow-up helped sustain engagement and identify potential technical issues or motivational challenges.

#### Data management with mobile and web-based applications

Following informed consent, demographic and medical data were recorded into the web-based clinical portal and linked to the patient’s unique identifier within the mobile application. A pediatric pulmonologist provided instructions to both children and their caregivers on proper spirometer use, including managing the Bluetooth connection, performing the pre-measurement calibration, and navigating the Android-based mobile application during spirometry sessions.

The mobile app calculated key spirometry parameters, including forced vital capacity (FVC), forced expiratory volume in one second (FEV_1_), FEV_1_/FVC ratio (Tiffeneau index: Tiff), peak expiratory flow (PEF), and mean forced expiratory flow between 25% and 75% of FVC (FEF_25–75_). Predicted values and corresponding percentages were derived based on the Global Lung Function Initiative (GLI) reference standards (31). According to ERS/ATS guidelines (30), the acceptance criteria required that the difference between the largest and the next largest FEV_1_ and FVC values be less than 0.1 L in three measurements per session.

Results were instantly transmitted to a clinical cloud system for real-time review by clinicians, enabling continuous monitoring and timely interventions. The web portal available to clinicians provided secure access to patient data, including graphical displays of previous spirometry measurements, an automated alert system for abnormal values, integrated messaging tools (e.g., push notifications and email), and comprehensive measurement histories. Timestamps were automatically created in the log-files for each measurement activity. This infrastructure enabled continuous remote monitoring and facilitated more proactive and individualized patient follow-up.

### Statistical analysis

The sample size estimation was based on a significance test for the Pearson correlation coefficient. The test was designed with 80% power, an expected effect size of ρ = 0.7, and a one-sided alternative hypothesis (positive correlation), using a commonly accepted significance level of 5%. The calculation yielded a minimum required sample size of 11. This analysis was performed using G*Power (version 3.1.9.7, Universität Düsseldorf, Germany).

Agreement between clinical and home spirometry measurements was primarily evaluated using Bland–Altman analyses (32), with limits of agreement calculated as the mean difference ± 1.96 standard deviations (SD) and accompanied by 95% confidence. Pearson’s correlation coefficients were calculated to assess linear associations, acknowledging that correlation does not imply agreement. These analyses were conducted for both absolute and percentage predicted spirometry values to assess measurement consistency across parameters.

The association between the number of home spirometric measurements and the number of reliable FEV_1_ and FVC estimates was analyzed using Pearson’s correlation test. As the study involved repeated spirometric measurements per subject over time, introducing both within-subject and between-subject variability, a mixed ANOVA approach with an interaction term was applied. The sphericity test failed; therefore, the Greenhouse–Geisser correction was used. Statistical analyses were performed using SigmaPlot for Windows (version 15, Systat Software, Inc., Chicago, IL, USA), and mixed ANOVA was performed using IBM SPSS Statistics version 29.0.0 (Build 241), with significance set at *p* < 0.05. All reported *p*-values are two-sided.

## Results

The demographic and clinical characteristics of the children are summarized in Table 1, including gender distribution, age, asthma severity and duration, and allergen sensitization. Most participants had mild persistent asthma and were on varying treatment regimens. The majority experienced at least one exacerbation requiring medical consultation in the past year.

**TABLE 1:** Demographic data and clinical characteristics of the children involved in the study.

Demographic data		
Gender	Female/male	8/3 (0.72)
Age (years)	Mean ± SD [min–max]	12.9 ± 3.1[8–17]
Body mass (kg)	Mean ± SD [min–max]	49.5 ± 18.5 [25–75]
Height (cm)	Mean ± SD [min–max]	153.5 ± 14.4 [130–174]
BMI (kg/m^2^)	Mean ± SD [min–max]	20.5 ± 5.7 [13.9–32.9]
Overweight/obesity (IOTF criteria > 25)		3/11 (0.27)
Age at diagnosis of asthma	Mean [min–max]	4.72 [1–11]
Duration of asthma (years)	Mean [min–max]	8.11 [4–15]
**Atopy-allergy**		
**Prick test or serum IgE (positive for inhalative allergens)**		
	House dust mite	6/11 (0.54)
	Pollen	7/11 (0.63)
	Mold	1/11 (0.09)
	Dog dander	3/11 (0.27)
	Cat dander	3/11 (0.27)
Atopic dermatitis		1/11 (0.09)
Allergic rhino-conjunctivitis		8/11 (0.72)
Food allergy		2/11 (0.18)
**Treatment (according to GINA 2024)**		
SABA (as required)	(Step 1)	0/11
ICS only	(Step 2)	3/11 (0.27)
LTRA only	(Step 2)	2/11 (0.18)
Combined ICS + LTRA	(Step 3)	2/11 (0.18)
Combined ICS + LABA/RABA	(Step 3)	2/11 (0.18)
Combined LTRA + ICS + LABA/RABA	(Step 4)	2/11 (0.18)
**Severity (GINA level)**		
Intermittent (level 1)		0/11
Mild persistent asthma (level 2)		9/11 (0.82)
Moderate persistent (level 3)		2/11 (0.27)
Severe persistent (level 4)		0/11
**Asthma control**		
> 1 medical consultation for asthma exacerbation in previous year		10/11 (0.91)
> 1 hospitalization for asthma in the previous year		1/11 (0.09)
> 1 hospitalization in intensive care for asthma, ever		0/11
Control according to GINA score	Well controlled	5/11
	Partly controlled	6/11 (0.54)
	Uncontrolled	0/11 (0.45)
ACT score < 20 (uncontrolled)	[min–max]	3/11 (0.27) [11–19]

**FIGURE 2:**
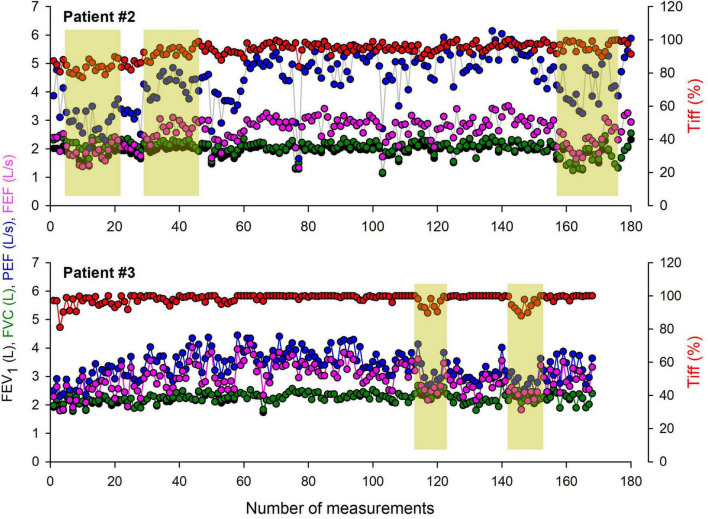
Time series of home spirometry parameters recorded over a one-year monitoring period in two representative children with moderate asthma. The graphs illustrate key respiratory function parameters, including forced vital capacity (FVC), forced expiratory volume in the first second (FEV_1_), FEV_1_/FVC ratio (Tiffeneau index), peak expiratory flow (PEF), and forced mid-expiratory flow (FEF_25–75_). Periods requiring specialist intervention are highlighted, indicating clinical deterioration, symptom exacerbation, or significant deviations from baseline values.

A time series of home spirometric parameters obtained in two representative children with moderate asthma are demonstrated in Figure 2. Relatively stable periods supplying reproducible values in the recorded outcomes are interrupted by temporary deteriorations in FEV_1_, FVC, PEF, and FEF_25–75_ in both children.

**FIGURE 3:**
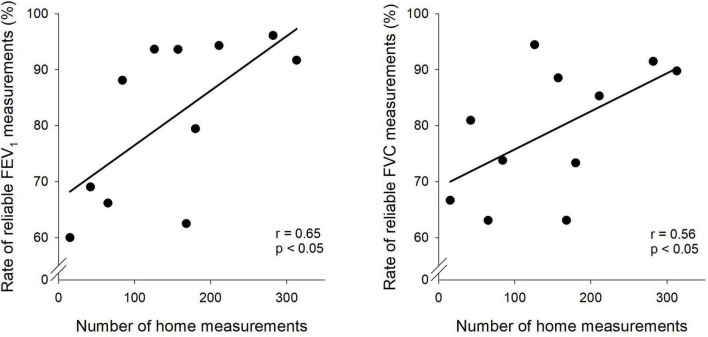
Association between home spirometry frequency and measurement reliability. Scatter plots depict the relationship between the number of home spirometry measurements and the reliability of forced expiratory volume in one second (FEV_1_) and forced vital capacity (FVC) measurements.

Figure 3 demonstrates the relationships between the number of home spirometric measurements performed by the children and the number of reliable estimates for FEV_1_ and FVC based on ERS/ATS criteria. A positive correlation was observed for both parameters, with reliability increasing as the number of home measurements increased (FEV_1_: *r* = 0.65, *p* < 0.05; FVC: *r* = 0.56, *p* < 0.05).

**FIGURE 4:**
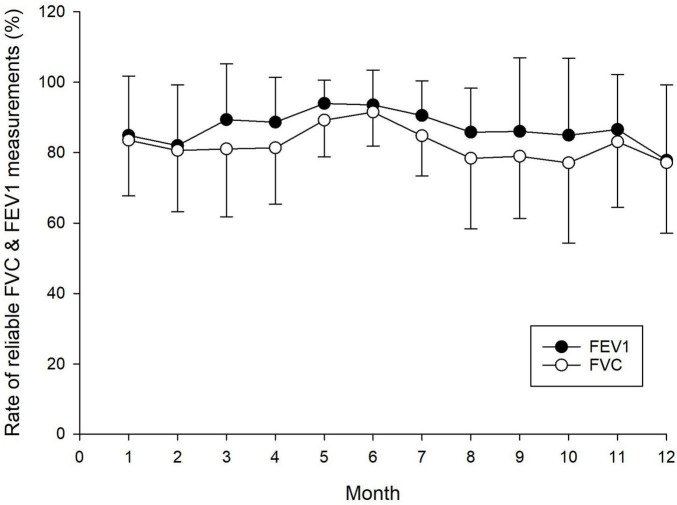
Reliability of home spirometry measurements over a 12-month period, showing the rate of reliable forced expiratory volume in one second (FEV_1_) and forced vital capacity (FVC) measurements. Each data point represents the mean and standard deviation of reliable FEV_1_ and FVC measurements.

Figure 4 illustrates the rate of reliable FEV_1_ and FVC measurements over the 12-month period. No significant interaction effect was observed, indicating no differential change over time between subjects. Overall, no statistically significant difference was detected in the shape of the trends. The between-subjects effect was also not significant, while the within-subject effect (i.e., the effect of time) yielded the lowest *p*-value but did not reach statistical significance at the 5% level.

**FIGURE 5:**
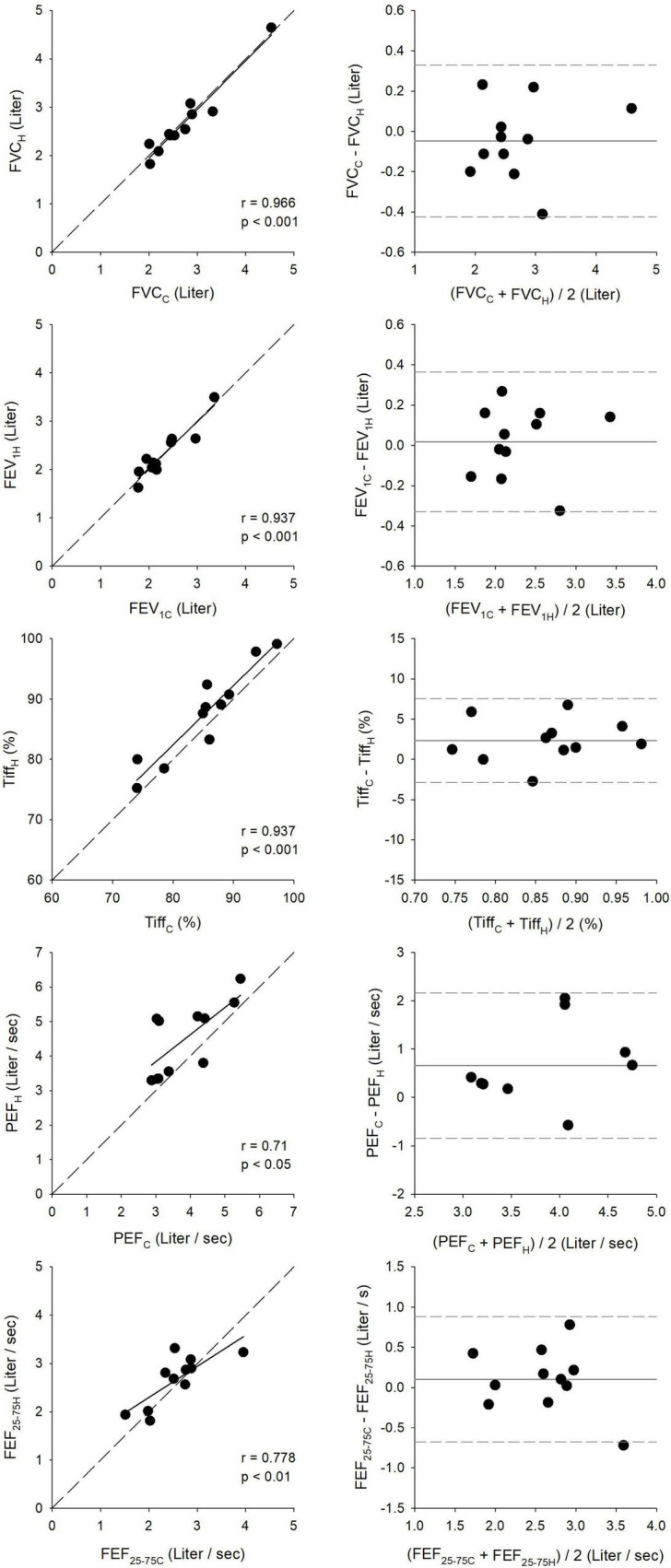
Correlation and agreement between clinical (C) and first home (H) spirometry measurements. The left panels show correlations between key respiratory parameters measured during clinical visits and first home assessments, including forced expiratory volume in 1 s (FEV_1_), forced vital capacity (FVC), peak expiratory flow (PEF), and forced mid-expiratory flow (FEF_25–75_). The right panels present Bland–Altman plots, illustrating agreement and measurement consistency between clinical and home recordings.

Relationships between spirometry outcomes obtained under pulmonologist supervision in the lung function laboratory and those derived from the first home measurement are demonstrated in Figure 5. Bland–Altman analyses revealed good agreement between the clinical and first home measurements, with narrow limits of agreement for FVC (0.75 L), FEV_1_ (0.69 L), and Tiff (10.0%) and somewhat wider limits of agreement for PEF (3.0 L/s) and FEF_25–75_ (1.6 L/s). Statistically significant correlations were observed between the clinical and home recordings, with the strongest correlation for FVC (*r* = 0.966, *p* < 0.001) and the least robust associations for PEF (*r* = 0.71, *p* < 0.05).

**FIGURE 6:**
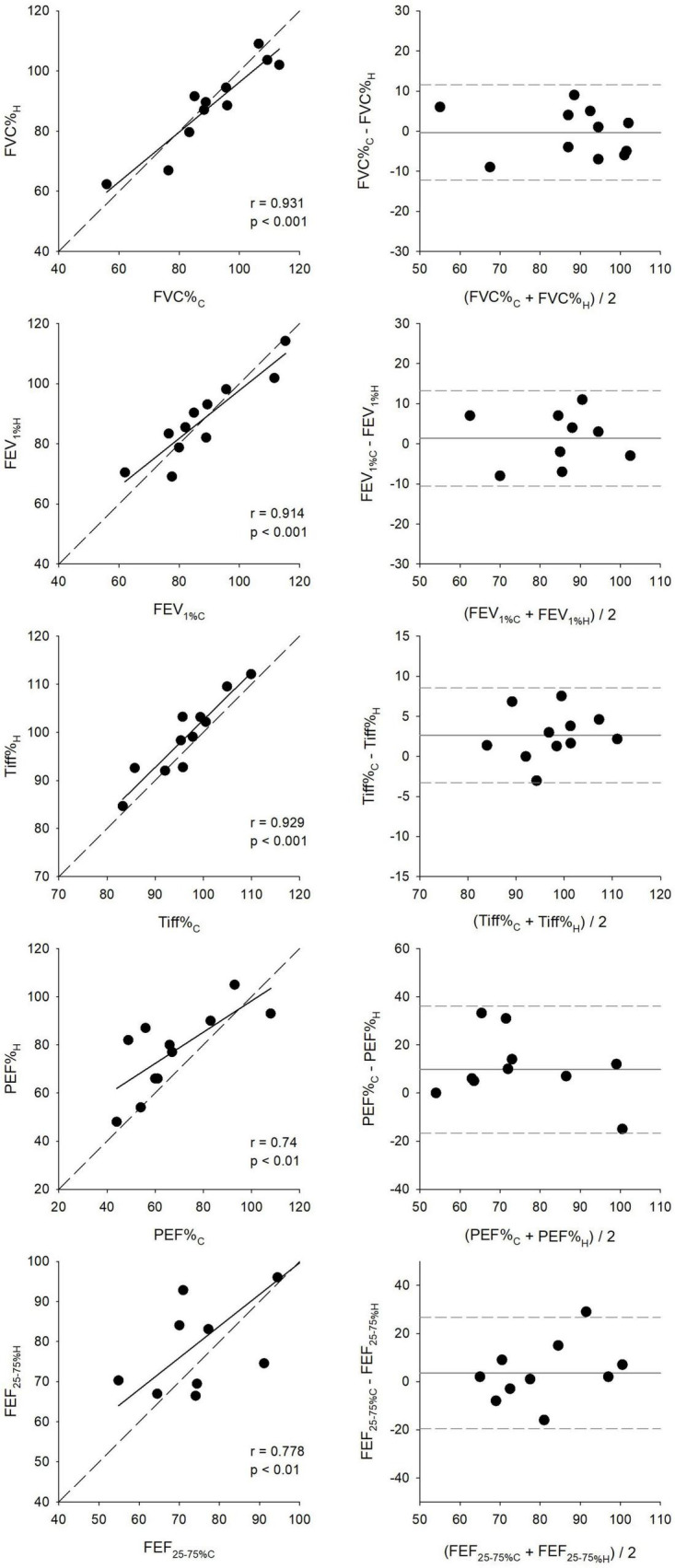
Scatter plots compare key respiratory parameters between clinical (C) and first home (H) spirometry measurements, including the percentage of forced vital capacity (FVC%), forced expiratory volume in the first second (FEV_1_%), FEV_1_/FVC ratio (Tiffeneau index, Tiff%), peak expiratory flow (PEF%), and forced mid-expiratory flow (FEF_25–75%_). All values are expressed as percentages of the predicted values set by the Global Lung Function Initiative (GLI). Correlation coefficients (*r*) and significance values (*p*) indicate the level of agreement between clinical and home measurements. Bland–Altman plots assess measurement consistency by displaying the mean difference between clinical and home values against their averages.

Expressing the spirometric parameters as percentage predicted values exhibited similar trends to those observed for their absolute values, as demonstrated in Figure 6. The narrowest limits of agreements were obtained by the Bland–Altman analyses for Tiff% (11.8%) and were intermediate for FEV_1%_ (23.7%) and FVC% (23.8%), and were the widest for FEF_25–75%_ (46.3%) and PEF% (52.7%). FVC% showed the strongest (*r* = 0.93, *p* < 0.001), while PEF% demonstrated the weakest correlation (*r* = 0.74, *p* < 0.05).

Ninety-five percent confidence intervals for the bias and limits of agreement for all key parameters, in both absolute and percentage predicted values, are presented in Table 2.

**TABLE 2:** Bland–Altman analysis of agreement between clinical and first home spirometry measurements, including bias, limits of agreement, and their 95% confidence intervals (CI).

Parameter	Bias (CI)	Lower limit (CI)	Upper limit (CI)
FVC (L)	0.048 (−0.180, 0.083)	−0.426 (−0.654, −0.198)	0.329 (0.102, 0.557)
FVC%	−0.36 (−4.50, 3.78)	−12.26 (−19.43, −5.09)	11.54 (4.36, 18.71)
FEV_1_ (L)	0.017 (−0.103, 0.138)	−0.329 (−0.538, −0.121)	0.363 (0.155, 0.572)
FEV_1_%	1.36 (−2.77, 5.49)	−10.50 (−17.65, −3.35)	13.23 (6.08, 20.38)
Tiff (%)	0.023 (0.005, 0.042)	−0.029 (−0.060, 0.003)	0.076 (0.044, 0.107)
Tiff%	2.65 (0.59, 4.70)	−3.25 (−6.81, 0.30)	8.55 (4.99, 12.11)
PEF (L/s)	0.657 (0.134, 1.180)	−0.846 (−1.751, 0.06)	2.160 (1.254, 3.065)
PEF%	9.75 (0.57, 18.92)	−16.62 (−32.51, −0.73)	36.11 (20.22, 52.00)
FEF_25–75_ (L/s)	0.100 (−0.171, 0.372)	−0.679 (−1.149, −0.210)	0.880 (0.410, 1.350)
FEF_25–75_%	3.55 (−4.51, 11.61)	−19.62 (−33.58, −5.66)	26.71 (12.75, 40.67)

## Discussion

The present study evaluated the feasibility of home lung function assessments in a vulnerable pediatric population with asthma bronchiale, focusing on the reliability, accuracy, and consistency of home-based spirometric measurements. Our results demonstrate a high acceptance rate in obtaining home spirometry parameters (> 60%) and generally stable lung function over the 12-month study period, with occasional temporary declines in key lung function outcomes. A positive correlation was observed between the number of home spirometry measurements and the reliability of FEV_1_ and FVC estimates. Throughout the 12-month period, the rate of reliable FEV_1_ and FVC measurements remained stable, with no significant temporal effects. Comparisons between clinical and initial home spirometry measurements showed strong correlations and good agreement, particularly for FVC and FEV_1_. When expressed as percentage predicted values, similar trends were observed, with FVC% exhibiting the strongest correlation and PEF% the weakest. The narrowest limits of agreement were found for the Tiffeneau index, while PEF% and FEF_25–75%_ displayed the greatest variability.

In the present study, significant correlations were observed between the number of home measurements and the reliability of spirometry results in children with asthma (Figure 3). Accordingly, an increased frequency of home spirometry measurements in asthmatic children increases patient familiarity with the procedure and minimizes potential measurement errors, thereby leading to improved accuracy and reliability of spirometric outcomes (13). This finding is in line with the results of earlier studies emphasizing a positive correlation between the regularity of home measurements and the reliability of spirometry results, and underscoring the necessity for frequent assessments to augment the accuracy of monitoring processes (33).

The reliability of measurements in this study was defined based on ATS/ERS acceptance criteria (30). Accordingly, at least three technically acceptable spirometry tests were required for each child, ensuring that the two largest FVC and FEV_1_ values differed by no more than 0.1 liter. The rate of reliable FVC and FEV_1_ measurements during home spirometry was approximately 80% and remained stable throughout the 12-month follow-up period (Figure 4). Notably, a significant proportion of home measurements met the acceptance criteria established by international guidelines (30). This performance is substantially higher than that reported in adults with asthma (22) and in children with acute asthma exacerbations (34). These findings underscore the feasibility of telemedical lung function monitoring through home spirometry, provided that children receive adequate training and parental supervision (13, 35–37). The high technical reliability of home-based spirometry in pediatric asthma presents a promising opportunity for enhanced disease management, potentially reducing the need for frequent specialist visits while minimizing associated risks, such as infection exposure and disruptions to daily activities (38).

Strong correlations and good agreement were observed between key spirometric parameters measured in clinical and home settings, whether expressed as absolute values or as percentages of predicted values (Figures 5, 6). The mean differences for FEV_1_ and FVC were within the Minimal Clinically Important Difference limits of 100–200 mL (or 5–10% of predicted values) (39). These findings highlight the potential of telespirometry for remote lung function monitoring in children with asthma. While strong agreement was observed for FVC and FEV_1_, the wider limits of agreement for PEF and FEF_25–75_ suggest poorer reproducibility. These parameters are known to be more effort-dependent and technically variable, particularly in pediatric populations (40–42). Therefore, although trends in PEF and FEF_25–75_ may still offer useful context, they should be interpreted cautiously for distant clinical decision-making.

Interestingly, Tiff and PEF values measured at home were higher than those obtained in the clinical environment using the same handheld spirometer. This discrepancy may be attributed to comprehensive patient education, which ensured accurate home measurements. Furthermore, these findings suggest that home spirometry may yield more reliable and representative lung function data, as children perform the tests in a familiar, stress-free environment, potentially minimizing anxiety or the white-coat effect commonly observed in clinical settings. The absence of external pressures from a clinical environment or physician presence may enable children to perform spirometry in a more relaxed and natural manner, leading to consistently higher Tiff and PEF values in home assessments. These results are in accordance with earlier findings demonstrating strong correlations and good agreements between spirometric outcomes measured in clinical and home settings (22, 43).

A methodological limitation of the present study is the relatively small number of children included in the follow-up of lung function via telespirometry. This can be attributed to the technically demanding measurement conditions and the extended study duration of one year. Nevertheless, the data analyses yielded clear and statistically robust results, demonstrating convincing correlation coefficients and rational limits of agreement. Thus, the inclusion of this pediatric population was sufficient to draw well-founded conclusions on the reliability, accuracy, and consistency of home-based lung function measurements with high confidence. The relatively small sample size reflects the feasibility-focused design of the study, consistent with accepted practices for pilot investigations. Despite this limitation, the statistically significant correlations, as well as the consistent reliability outcomes observed, provide meaningful support for the feasibility of home telespirometry in pediatric asthma. Another limitation is the absence of a control group, such as standard hospital-based monitoring. Since our primary aim was to assess the technical performance and reliability of home spirometry rather than clinical outcomes, a control group was not incorporated by design.

In summary, the results of the present study support the feasibility and reliability of home spirometry for long-term respiratory monitoring in asthmatic children with digital literacy. The positive correlation between the rate of reliable home spirometry and the number of measurements demonstrates the importance of practice, besides training. The strong agreement with clinical measurements suggests that home spirometry could serve as a valuable tool for remote patient monitoring, potentially improving asthma control and management in this particularly vulnerable population. Moreover, the stability of reliability over time indicates that frequent at-home monitoring can provide consistent and clinically useful data, reinforcing its potential integration into routine pediatric patient care. Thus, the integration of telespirometry into asthma management for children has the promise to represent a significant advancement in healthcare delivery. Telespirometry enables long-term monitoring of spirometric data over weeks or months, tailored to the patient’s clinical needs. It not only facilitates timely intervention during exacerbations but also empowers patients and their families to take an active role in managing the condition.

## Data Availability

The raw data supporting the conclusions of this article will be made available by the authors, without undue reservation.
